# Depressed and Socioeconomically Disadvantaged Mothers’ Progression Into a Randomized Controlled Mobile Mental Health and Parenting Intervention: A Descriptive Examination Prior to and During COVID-19

**DOI:** 10.3389/fpsyg.2021.719149

**Published:** 2021-08-12

**Authors:** Kathleen M. Baggett, Betsy Davis, Elizabeth A. Mosley, Katy Miller, Craig Leve, Edward G. Feil

**Affiliations:** ^1^Mark Chaffin Center for Healthy Development, Georgia State University, Atlanta, GA, United States; ^2^Oregon Research Institute, Eugene, OR, United States

**Keywords:** mobile intervention, remote coaching, maternal depression, parenting, infant, COVID-19

## Abstract

Infants of low-income and depressed mothers are at high risk for poor developmental outcomes. Early parenting mediates infant experiences from birth, and early intervention can support sensitive and responsive parent practices that optimize infant outcomes via promoting developmental competencies. However, low-income and depressed mothers experience substantial challenges to participating in early intervention. They also have extremely limited access to interventions targeting depression. Interventions targeting maternal depression and parent practices can improve maternal and infant outcomes. Mobile internet-based interventions overcome numerous barriers that low-resource mothers face in accessing home-based interventions. Pandemic-related stressors likely reduce family resources and exacerbate distress of already heavily-burdened mother-infant dyads. During crises such as the COVID-19 pandemic, evidence-based remote coaching interventions are paramount. This article reports on a mobile intervention for improving maternal mood and increasing parent practices that promote infant development. An ongoing randomized controlled trial study provided a unique opportunity to monitor progression from referral to intervention initiation between two groups of depressed mothers: those prior to the pandemic and during the pandemic. The study also examines mother and infant characteristics at baseline. The sample consisted primarily of Black mothers experiencing extreme poverty who self-referred to the study in a large southern city, which is one of the most income disparate in the United States. Prior to the pandemic, 97% of study participants successfully progressed from consent to intervention, as compared to significantly fewer–86%–during the pandemic. Mother-infant dyads during COVID-19, as compared to those prior to COVID-19, displayed similar pre-intervention demographic characteristics and intrapersonal characteristics.

## Introduction

The individual and societal costs of depression are enormous, with depression disproportionately affecting women as a leading cause of disability globally (World Health Organization, 2020). Compared to any other time during the life course, women are more likely to experience depression and anxiety in the first year postpartum (Miller and LaRusso, 2011). Women in the United States, who are socioeconomically disadvantaged and identify as a non-dominant culture group member^1^, experience depression at a rate of 4–5 times higher than the national average (National Center for Health Statistics, 2016). The burden of depression is borne by mothers as well as their children. Maternal depression can interfere with parenting and compromise children’s social-emotional, communication, and cognitive development (Mental Health America, 2018; Goodman et al., 2020; Rogers et al., 2020) interfering with sensitive and responsive parenting practices that are essential for healthy child development (Center on the Developing Child at Harvard University, 2016). Consequently, integrated interventions that target both maternal depression and sensitive and responsive parent practices are crucial to initiate as early as possible postpartum to foster maternal and child health and development (Goodman and Garber, 2017). Hence, multiple professional organizations have called for women to receive depression screening and referral to intervention during the first year postpartum (American Academy of Pediatrics, and American College of Obstetricians and Gynecologists, 2017).

Although universal screening and referral to address depression is recommended by the American Academy of Pediatrics (AAP) and the American College of Obstetricians and Gynecologists (ACOG) (Rafferty et al., 2019), most women do not receive screening and appropriate intervention (Baker et al., 2020). Moreover, Black and Latinx women are far less likely receive intervention as compared to white women (Grote et al., 2007). In the United States, white women are more than twice as likely to use mental health services as compared to Black or Latinx women (Substance Abuse and Mental Health Services Administration, 2015), consistent with the general pattern in which white persons are significantly more likely to receive mental health services when compared to individuals of non-dominant cultures. This disparity exists after controlling for multiple intraindividual characteristics (i.e., factors within the individual) including symptom severity (Ramos and Chavira, 2019).

Depressed mothers, as compared to those who are not depressed, are less likely to enroll and engage in Maternal, Infant and Early Childhood Home Visiting interventions (Maternal, Infant, and Early Childhood Home Visiting Technical Assistance Coordinating Center, 2015). Due to structural and systemic biases, depressed racial and ethnic minority individuals are more likely to be socioeconomically disadvantaged than depressed white individuals in the United States (Bailey et al., 2017). Another contributor to disparities in intervention access in the United States is the redistribution of wealth that has shifted social safety net resources away from the very poor (Moffitt, 2015). Research is not immune to problems of inequity in intervention access. Systemic and structural barriers to recruitment of participants from non-dominant cultures into clinical efficacy and effectiveness trials include distrust in research as well as costs and logistics that impede participation (Durant et al., 2014; Oh et al., 2015). While inclusion of individuals of minority racial and ethnic backgrounds and those who are socioeconomically disadvantaged has increased over the past 40 years (Polo et al., 2019), individuals of European descent continue to be the most prominently represented group. When non-dominant groups are represented in samples, studies often fail to report racial/ethnic characteristics sufficiently to understand intervention engagement or to benefit these populations (Williams et al., 2013). Studies often do not examine racial/ethnic background as moderators of treatment effects, limiting understanding of effectiveness of intervention within these populations (Geller et al., 2011).

In general, recruitment of depressed participants into clinical trials is difficult, and lower resourced individuals are not well-represented within these efforts. In the United States, large numbers of individuals from non-dominant cultures are under- or uninsured, contributing barriers to health-related service receipt (Lillie-Blanton and Hoffman, 2005) as well as inclusion in clinical trials (Cho et al., 2018). Given the ubiquity of digital technologies, mobile health interventions increase intervention access for those who are traditionally missed (Anderson-Lewis et al., 2018). However, currently published studies are extremely limited with regard to reporting successful recruitment and progression into mobile health interventions among non-dominant culture mothers, who are depressed and experiencing significant socioeconomic disadvantage. The lack of systematic studies that focus on mobile recruitment to mobile intervention engagement in this vulnerable group during non-pandemic times is particularly concerning given the pressing need for such interventions during pandemic.

Studies on the effects of COVID-19 on postpartum mothers and their infants are just beginning to emerge. Mothers who are poor and minoritized are disproportionately affected by COVID-19. Additionally, the health care systems that serve them have been severely affected by COVID-19, likely contributing to maternal stress. Two systematic reviews document significantly increased stress for mothers postpartum during the COVID-19 pandemic (Hessami et al., 2020; Chmielewska et al., 2021). The largest review, including 40 pooled studies, showed significantly higher levels of depression during pandemic as compared to prior pandemic among postpartum women (Chmielewska et al., 2021). Pandemic increases in financial stress and isolation-induced loneliness are associated with increased depression, and effects are worse for low-income women (Perzow et al., 2021).

We are currently conducting a clinical trial evaluating the efficacy of an integrated internet-based parenting and depression intervention. The intervention is designed to reduce maternal symptomatology and increase sensitive and responsive parenting practices of mothers to optimize infant outcomes. This study, which takes place within the urban core of a large southern United States city, provides a valuable context in which to describe referral and progressive steps to intervention engagement among two groups of mothers: those prior to and during the pandemic. Results have the potential to provide information about improving access to intervention in general and during pandemic for mother-infant dyads affected by maternal depression, poverty, and minoritization that hinders service access.

This current study describes the progression into intervention for mothers before and during the COVID-19 pandemic. Because researchers may succeed or fail in engaging mothers at multiple points in the recruitment process, we examine the relative success at several points between initial referral and engagement in the intervention as described more specifically below. The current study used data from the ongoing trial spanning a 22-month period prior to the COVID-19 pandemic and a 10-month period following the pandemic to describe referral to and recruitment into the intervention. Because most of the pandemic-involved sample did not have sufficient time in the study to provide an opportunity to complete the entire intervention at the time of this report, we focus on completion of the first intervention session as an indicator of connection to intervention. The trial also provides a unique context within which to compare preliminarily potential pandemic versus non-pandemic group differences for intraindividual characteristics associated with adverse maternal and infant outcomes. The questions we address are:

(1) Do study mothers, whose study experience occurs during pandemic, as compared to mothers prior to the pandemic, show differential success in connecting to the intervention as defined by: (a) percent referred; (b) percent screened for eligibility; (c) percent eligible; (d) percent consented; and ultimately, (e) percent of mothers who initiate intervention?

(2) Do study mothers and infants with pandemic experience, as compared to those prior to the pandemic, differ on intraindividual characteristics that are associated with adverse maternal and infant outcomes, including maternal depression symptom severity, parenting self-efficacy, maternal knowledge of infant social-emotional and communication development and its promotion, and infant negative affect?

## Materials and Methods

Our recruitment efforts were aimed at generating a sample inclusive of mothers from non-dominant cultures, who were experiencing socioeconomic disadvantage and elevated maternal depressive symptoms (Baggett et al., 2020a). In so doing, two groups of mother-infant dyads emerged, those whose study experience was prior to the COVID-19 pandemic and those whose study experience occurred during the pandemic. Inclusion criteria were intended to produce a sample of mother-infant dyads, in which infants were at elevated risk for poor social emotional and communication development as a function of maternal depression and adverse mother-infant interactions that exacerbate the detrimental effects of poverty. Prior to initiating human subject activity, all study procedures were approved by the Georgia State University IRB. For inclusive sampling, recruitment strategies included community agency referrals, research staff outreach visits to community agencies and community events, and maternal self-referral. Potentially eligible women were contacted by research staff who described the project, conducted eligibility screening, and obtained informed consent. Consented participants were randomized into one of two parallel intervention arms: (1) Mom and Baby Net (MBN) or (2) Depression and Developmental Awareness (DDAS). MBN is a 14-session, coach-facilitated, online intervention that teaches mothers both cognitive-behavioral strategies to reduce depressive symptoms and specific skills for engaging with their infants to promote infant social communication competencies. DDAS is an informational program designed to improve maternal awareness of depression and understanding of infant developmental milestones. The MBN is a skill-based program designed to promote parental competencies to address affective symptoms and interact positively with their infants. In contrast, DDAS is an informational program that provides relevant content but does not focus on skill acquisition. The two mobile interventions were identical regarding number of sessions, session length, and delivery mechanisms. For more information about the interventions, see Baggett et al., 2020b.

For this report, we focus on the study period January 2018 through May 2021, which provided sufficient study time for participants prior to and during pandemic to have the opportunity to progress from consent to completion of the first remote coaching session. Outcomes between the two groups included description of the following: percent of referred, screened, eligible, consented, and completion of an initial remote coaching session to connect with intervention. We also examined between-group intraindividual characteristics that present risk for adverse mother-infant interactions and poor infant social communication development. These factors included: depression symptom severity, parent sense of competence, knowledge about infant social-communication development and its promotion, and infant negative affect.

### Referral

Whereas maternal online self-referral was the primary referral mechanism prior to the pandemic (Baggett et al., 2020a), it was the exclusive referral mechanism during the pandemic. The project maintained a self-referral mechanism through its website, which provided the following: (1) access to a brief video describing the intervention program; (2) information about the project team; (3) depression screening; and (4) a form for providing contact information to research staff. Prior to the pandemic, to promote awareness of the online self-referral mechanism, the research team posted information on local community agency websites, social media platforms, and in print material available at local community agencies. Additionally, community agency staff provided referrals and our research staff visited community agencies and attended community events, such as resource fairs, at which service agencies advertise their programs. Staff provided interested women with information about the intervention project, screened for inclusion criteria, and referred mothers to the project coordinator for enrollment.

### Sample

Participants referred were mothers of infants aged 0–12 months (***N*** = 438). Mothers were included in the study sample if they had a score of 3 or more on the Patient Health Questionnaire-2 (PHQ-2) (Kroenke et al., 2003) at screening, were a minimum of 18 years old, spoke English, and lived in the local metropolitan area of a large southern city in the United States. Exclusion criteria were: history of psychotic symptoms, residence in homeless or domestic violence shelter, mother or infant receiving intensive medical treatment, and not having permanent legal guardianship of infant. Because there were no significant mean differences by the pandemic-exposed and non-pandemic group (except for infant age), demographic characteristics are presented for the overall sample in Table 1. Due to COVID-19 research delays, dyads remained on the waitlist for 2.5 months, during which time COVID-19 study procedures were submitted to and reviewed and approved by the IRB. Consequently, infants in the pandemic-exposed group were on average 2.5 months older than infants in the non-pandemic group.

**TABLE 1:** Demographic characteristics of the pre-intervention sample.

**Variable (*N* = 181)**	**Value**
Maternal age in years, mean (SD); range	28.17 (5.83); 18.54–46.09
Child age in months, mean (SD); range	6.04 (2.85); 0.59–11.89
Number of children in the home, mean (SD); range	2.63 (1.41); 1.00–6.00
Maternal race (Black)% (*n*)	87.84% (159)
Maternal ethnicity (Latinx),% (*n*)	2.76% (5)
Maternal education (<college degree),% (*n*)	83.98% (152)
Maternal income,% </= 125% Federal Poverty Guideline, (*n*)*	84.18% (149)*
No significant other, % (*n*)	73.48% (133)

*Four missing, so *n* = 177.

### Measures

To assess maternal progression from referral through successful recruitment into the study intervention, the following variables were documented by date of occurrence or disposition within the project database: (1) referred, (2) screened for eligibility, (3) eligibility, (4) completion of comprehensive pre-assessment, (5) completion of an intervention orientation session, and (6) completion of the first intervention session. The PHQ-2 was administered online to screen for depression with the established criteria of a score of 3 or higher defined as a positive depression screen. The PHQ-2 is an efficient and well-established measure with strong psychometric characteristics for identifying individuals with depression (Kroenke et al., 2003). At pre-intervention assessment, participants completed a demographic questionnaire to facilitate characterization of the sample for mother’s age, ethnicity, race, educational level, income, significant relationship status, number of children in the home, and infant age in months.

Participant intrapersonal risk characteristics were assessed at pre-intervention. The Patient Health Question-9 (PHQ-9) was administered to assess depression severity (Wisner et al., 2013). The PHQ-9 possesses strong psychometric properties for assessing depression severity; a score at or above 20 is suggestive of severe depression (Kroenke et al., 2001). Participants were also administered the Knowledge of Infant Social communication Development and Competency Promotion, which has demonstrated high internal consistency and sensitivity to intervention change (Feil et al., 2020). The Parenting Sense of Competence was designed to measure parent self-efficacy. It is a 17-item, Likert type scale demonstrating adequate psychometrics (Johnston and Mash, 1989). The Infant Behavior Questionnaire-Very Short Form (IBQ-R VSF) was used to assess infant negative affect. Psychometric properties of the negative affect scale have been examined with racially and economically diverse samples and are adequate (Leerkes et al., 2017).

### Analysis

**FIGURE 1:**
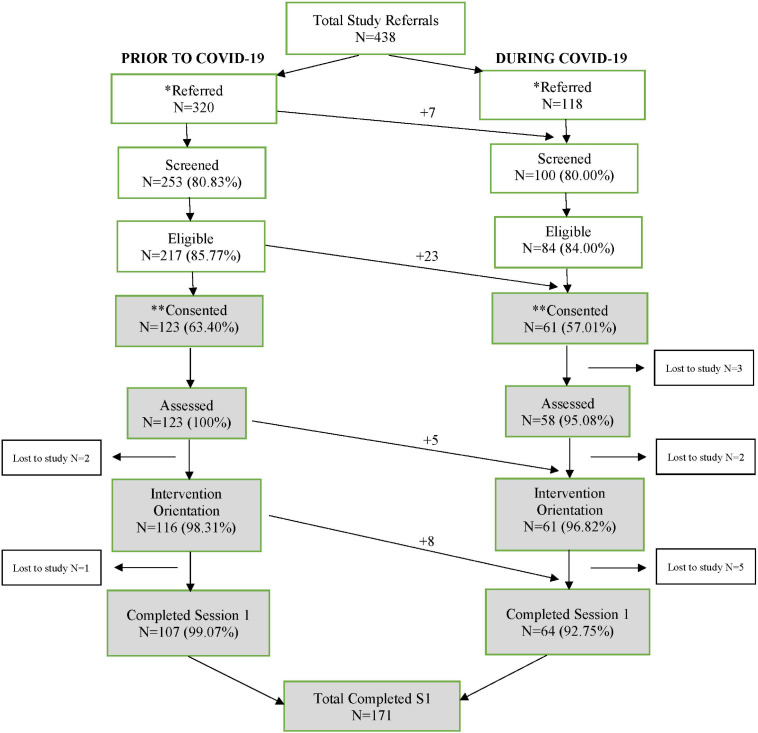
Referral to intervention engagement progression. Arrows from left to right signify participants moving from pre-pandemic experience into pandemic experience at each study progression point. *Prior to COVID, 320 mothers were referred over a 22-month period resulting in an average of 14.5 referrals per month, equivalent to less than an average of two referrals per day. During COVID, 118 mothers self-referred in response to a provider text blast. Over a 3-day period, 118 referrals are equivalent to an average of 39 mothers per day. Hence, mothers referred at a relative daily rate of 19.5 times higher during COVID as compared to non-covid. Referral was closed after this 3-day referral period. **It was not possible to consent all referred, screened, and eligible mothers during COVID because this number exceeded the number of open slots for targeted enrolment. Prior to COVID, a 2.43% of participants were lost to the study after consent as compared to 16.39% lost during pandemic. Hence, 6.75 times more mothers were lost to the study during the pandemic.

Using data collected between January 2018 and May 2021 on 438 mothers, we describe the progression of 320 mothers referred prior to the pandemic and 118 mothers referred during the pandemic through the six successive points into intervention: (1) referral, (2) screening, (3) eligibility determination, (4) consent to intervention trial, (5) completion of intervention orientation, and (6) completion of the first intervention session. It is important to note that, as displayed in Figure 1, some mothers who were referred prior to the pandemic, entered the pandemic experience at subsequent points of progression toward intervention. Following progression description, we then used individual samples *t*-tests to compare intraindividual characteristics mothers with no pandemic experience to mothers with pandemic experience. To control for Type 1 error, we held our examination to four multiple comparisons, providing 80% power to detect small between-group effects (*d* = 0.35 or above) for two-sided tests at *p* < 0.0125, with our lowest sample size of *n* = 174.

## Results

Prior to the pandemic (see Figure 1), 320 mothers were referred over a 22-month period resulting in an average of 14.5 referrals per month, equivalent to less than an average of two referrals per day. During COVID-19, 118 mothers self-referred in response to a single provider text blast. Over a 3-day period, the 118 referrals are equivalent to an average of 39 referrals per day. Hence, mothers referred at a relative daily rate of 19.5 times higher during COVID-19 as compared to before the pandemic. Prior to COVID-19, 2.43% of participants were lost to the study after consent as compared to 16.39% lost during the pandemic. Hence, 6.75 times more mothers were lost to the study during the pandemic as compared to prior. When viewing the number of mothers consented during the pre-pandemic period (*n* = 123), less the number of mothers who moved into COVID-19 progression before completing intervention (*n* = 13), 97% of mothers completed session. Within the COVID-19 progression, however, 61 mothers were consented, with 13 mothers moving into this progression before intervention connection (total *n* = 74). Of these, 64 (86%) of mothers completed session 1 during the COVID-19 pandemic.

Our second question focused on intrapersonal risk characteristics of mothers with study experience during pandemic as compared to those prior to pandemic. We conducted *t*-tests, holding examination to four comparisons for sufficient power to detect small effects as described in the Analysis section. As displayed in Table 2, there were no significant differences between the pandemic-exposed and non-pandemic group for maternal depression severity, parenting sense of competence, mother knowledge of infant social-emotional and communication development and its promotion, or infant negative affect.

**TABLE 2:** Intrapersonal risk characteristics by pandemic and non-pandemic group.

**Variable**	**Non-pandemic group mean**	**Pandemic group mean**	***t*-statistic**	***p*-value**
Depression severity	0.34	0.23	1.59	0.113
Parent sense of competence	49.03	46.72	1.18	0.240
Parent knowledge of infant social-emotional development and promotion	5.28	4.87	1.00	0.319
Infant negative affect	4.51	4.64	−0.72	0.470

## Discussion

The purpose of this study was to describe progression into intervention between two groups of mothers: those during the COVID-19 pandemic as compared to those prior to the pandemic. Mobile parenting interventions have the potential during pandemics to broaden access to crucial parenting and mental health supports for underserved communities by reducing instrumental, social, and psychological barriers. They can overcome the public health challenges (e.g., the need for service greatly exceeding capacity of providers), and they provide evidence-based interventions otherwise not available in many communities. To date, these interventions have demonstrated success in many areas, but have also been shown to experience challenges similar to community-based care such as difficulties with recruitment, engagement, and attrition (Lane et al., 2015; Laws et al., 2016; Anderson-Lewis et al., 2018). The COVID-19 emergency created new urgency and opportunity to examine the extent to which mobile parenting and mental health programs can address a sudden and broad breakdown in availability of and access to traditional delivery methods for parenting and mental health interventions. Health systems, including mental health systems, were profoundly affected by the substantial challenges of increased service demand due to elevated stressors and reduced availability of in-person services due to demand for social distancing (Hessami et al., 2020; Chmielewska et al., 2021; Perzow et al., 2021).

Regarding our first research question about progression into intervention, we found that from consent to intervention initiation, study mothers in the pandemic fell away from intervention at a rate of nearly seven times higher than study mothers prior to the pandemic. However, both pandemic and non-pandemic rates of successful progression into intervention were quite high with 97% of mothers pre-pandemic and 86% of pandemic-exposed mothers progressing successfully from consent through comprehensive pre-assessment and intervention orientation to completion of the first intervention session. These findings suggest that recruitment and engagement into a mobile parenting intervention is feasible during a prolonged public health emergency within one of the most income disparate cities in the United States, in which the sample was characterized by poverty, minoritization, low partner support, low education level, and depression. These findings compare very favorably to home visiting interventions, in which pre-pandemic progression from consent to receipt of one intervention sessions range from 56 to 97% with parents who are depressed and facing multiple adversities, least likely to successfully progress to receiving an intervention session (Maternal, Infant, and Early Childhood Home Visiting Technical Assistance Coordinating Center, 2015).

For our second research question, the finding of no differences between study participants with and without pandemic experience, relative to demographics and intraindividual characteristics, including depression severity, parenting knowledge of infant social-emotional development, maternal parenting stress, and infant negative affect—was surprising. However, it is not entirely inconsistent with existing available data. Among the two largest metanalytic studies of pandemic effects on postpartum women and their infants, one found significant pandemic effects on depression and the other did not. The study that focused on general health systems found significantly elevated depression during the pandemic (Chmielewska et al., 2021). However, the other, which focused on existing clinical mental health program patients, showed elevated but not significantly elevated symptoms from pre-pandemic to pandemic conditions (Hessami et al., 2020). Another study found that pandemic financial strain and social isolation were significant drivers of increased depression, especially for low-income women (Perzow et al., 2021). One possibility is that within our study, mothers with depression, who were connected to a supportive intervention early in the pandemic, may have experienced less isolation-induced distress and depression as compared with mothers in the general population who lack such supports. However, this remains to be determined by future study.

Several constraints of the present study and directions for ongoing research should be noted. First and foremost, this is a descriptive study in which participants were not randomized to participate during the pre-pandemic versus pandemic periods. Though an obvious point, it warrants mention because it necessitates caution in interpretation of the findings. It is likely that findings herein are at least partially attributable to pandemic disruptions and stressors. However, we cannot rule out the possibility that other independent factors accounted for lower rates of successful progression in the pandemic group. Moreover, we cannot know whether noted differences in progression of participants through the engagement process were attributable to differences in who chose to participate in the pandemic as compared to non-pandemic cohorts. Although the absence of observed differences in measured characteristics reduces this likelihood, it is possible that unmeasured differences contributed. Additionally, the inclusion criteria of speaking English limited our ability to recruit non-English speaking participants, which limits our ability to generalize these findings to non-dominant groups excluded by the language requirement. These groups could be considered and centered in future studies. Finally, at the time of this report, the data were not yet available to examine the extent to which the pandemic affected progression through the intervention. Therefore, questions regarding engagement in the interventions, including pandemic effects on attrition, dosage or adherence, time to complete, or relatedly mobile coach behavior and fidelity await further study.

## Conclusion

An ongoing randomized controlled trial afforded us the opportunity to examine the extent to which recruitment and engagement into a mobile parenting intervention would be robust to disruptions associated with the COVID-19 pandemic. Our specific case focused on engagement of depressed and socioeconomically disadvantaged mother-infant dyads into a postpartum intervention to reduce depression and promote sensitive-responsive parenting practices that optimize infant social-emotional and communication outcomes. Overall, our findings showed robust referral during pandemic and rates of successful progression into intervention that were at least as favorable as those reported in routine studies of home visiting intervention programs outside of pandemic. These results point to the importance of inclusive recruitment methods—in particular, online self-referral of mothers (see Baggett et al., 2020a for details on recruitment strategies). Moreover, women and infants enrolled in the trial did not differ, as a function of pandemic experience during the study, with regard to maternal depression severity, parenting self-efficacy, knowledge regarding infant social-emotional development, or infant negative affect. These findings suggest that the use of mHealth interventions hold significant promise as strategies for provision of mental health and parenting services during periods of widespread service disruption due to public health emergencies. Although it is too early to know how well these findings will generalize, or whether they will carry over to progression through the intervention phase, they point more broadly to the potential of mobile interventions to enable service delivery for a range of parenting and behavioral health needs during emergency situations.

## Data Availability Statement

The datasets presented in this article are not readily available because these data are part of an ongoing clinical randomized controlled trial. Anonymized data from this substudy can be made available on request. Requests to access the datasets should be directed to KB, kbaggett@gsu.edu.

## Ethics Statement

The studies involving human participants were reviewed and approved by Georgia State University IRB. The patients/participants provided their written informed consent to participate in this study.

## Author Contributions

KB and BD: conceptualization, methodology, and project administration. KM and EF: software. KB, BD, CL, and KM: formal analysis. KB, BD, and EF: investigation and resources. KB, BD, and EM: writing–original draft preparation and writing–review and editing. KB: supervision and funding acquisition. All authors have read and agreed to the published version of the manuscript.

## Conflict of Interest

KB, BD and EF are the developers of the InfantNet program, the original intervention platform on which the ePALS Mom and Baby Net program application is based. The remaining authors declare that the research was conducted in the absence of any commercial or financial relationships that could be construed as a potential conflict of interest.

## Publisher’s Note

All claims expressed in this article are solely those of the authors and do not necessarily represent those of their affiliated organizations, or those of the publisher, the editors and the reviewers. Any product that may be evaluated in this article, or claim that may be made by its manufacturer, is not guaranteed or endorsed by the publisher.

## Footnotes

1 Non-dominant culture members are those from disenfranchised, subordinated, and marginalized communities, for example, non-White and lower income.
